# Comparison between anterior segmental osteotomy versus conventional orthodontic treatment in root resorption: a radiographic study using cone-beam computed tomography

**DOI:** 10.1186/s40902-017-0131-3

**Published:** 2017-11-25

**Authors:** Bo-Yeon Hwang, Byung-Joon Choi, Baek-Soo Lee, Yong-Dae Kwon, Jung-Woo Lee, Junho Jung, Joo-Young Ohe

**Affiliations:** 1 Department of Oral and Maxillofacial Surgery, Graduate School, Kyung Hee University, Seoul, Republic of Korea; 2 Department of Oral and Maxillofacial Surgery, School of Dentistry, Kyung Hee University, Seoul, Republic of Korea; 3 Department of Oral and Maxillofacial Surgery, Kyung Hee University Dental Hospital, Seoul, Republic of Korea

**Keywords:** Anterior segmental osteotomy, Conventional orthodontic treatment, Root resorption

## Abstract

**Background:**

Patients who received orthodontic treatment are likely to have apical root shortening. It appears that external apical root resorption results from a combination of patient-related risk factors such as genetic influences, systemic factors, and orthodontic treatment-related factors. Regarding the fact that the anterior segmental osteotomy (ASO) has been known for its possibility of complementing external apical root resorption and of buffering periodontal problems, it has been the preferred treatment. However, the studies on the efficacy of ASO in preserving the root are not sufficient. In this study, we compared the amount of root resorption between the patients who only received orthodontic treatment and the patients who received orthodontic treatment with ASO.

**Methods:**

This study included 28 patients (the number of incisor = 198) who received orthodontic treatment with or without ASO. We categorize them into groups A and B by the type of orthodontic treatment (group A: conventional orthodontic treatment; group B: orthodontic treatment with ASO). Cone-beam computed tomographic and cephalometric evaluations were retrospectively performed on the radiographs taken for the diagnosis of the treatment before treatment and at the end of active treatment.

**Results:**

In group B, root resorption itself and its rate both turned out to have significantly lower than those in group A. Also, the change of incisal angle is significantly smaller in group B than in group A. On the other hand, in group A, the change of incisal angle was positively correlated with the change of AP (anteroposterior) position. In group B, the change of incisal angle was negatively correlated with the duration of the orthodontic treatment. In group B, amount of root resorption (mm) was positively correlated with the duration of the orthodontic treatment.

**Conclusions:**

The results show lesser root resorption and shorter treatment duration with ASO than with conventional orthodontic treatment. Therefore, if the indications are accurately determined, ASO can be an effective treatment option when the amount of root resorption is expected to be high, especially in late adults.

## Background

Anterior retraction is required for the improvement of facial forms in patients with bimaxillary protrusion or maxillary or mandibular protrusion, where the mouth is protruded due to skeletal or dental problems. When the deformities in these patients are corrected through orthodontic treatment, the treatment steps generally include retraction of the anterior teeth using the space created by extraction of the maxillary first premolars. However, this treatment can cause adverse effects such as limited improvements in the facial form, root resorption, severe retroclination of the anterior teeth, cleavage or perforation of the labial bone, insufficient retraction due to the lack of an anchoring force, and unnecessary downward shifts of the maxillary anterior teeth [1, 2].

In particular, adult patients exhibit limited physiological tooth movement because of the decrease in blood supply with age; therefore, long-term treatments are relatively more difficult [3]. This may be accompanied by poor periodontal health and social factors such as esthetics. Therefore, the demand for shorter treatment duration is increasing [4].

To overcome these limitations, space closure through anterior segmental osteotomy (ASO) after extraction of the premolar tooth is considered a desirable treatment option. Since the introduction of ASO by Cohn-stock in 1921 [5], it has been modified and developed by Wassmund [6], Cupar [7], Schuchardt [8], Wunderer [9], Bell and Condit [10], and Park and Hwang [11].

ASO results in an immediate improvement in the facial form, effectively eliminates excessive gingival visibility while smiling or the so-called “gummy smile,” and prevents the downward shift of the anterior teeth, which can occur during tooth retraction. In addition, it is feasible for patients with thin surrounding alveolar bone or thin tooth roots, in whom a rapid orthodontic force can cause root resorption and bone perforation or cleavage [12].

The rate of root resorption in patients who undergo ASO is speculated to be lower than that in patients who undergo orthodontic treatment only, although there are no direct studies on this topic. Thereby, in the present study, we compared root resorption between patients treated by conventional orthodontic treatment and those treated by ASO with orthodontic treatment.

## Methods

### Subjects

This study was performed from 2007 to 2013. We used 198 incisors who had undergone orthodontic treatment with or without orthognathic surgery after diagnosis with skeletal bimaxillary protrusion or maxillary or mandibular protrusion at the Department of Oral and Maxillofacial Surgery of Kyung Hee University Dental Hospital. The patients consisted of 5 males and 23 females, and their ages ranged from 19 to 29 years old at the time of the surgery (mean age of 22.2). They were divided into two groups (Table 1). All the patients have crowding and spacing of the dentition (< 3 mm) with Angle classification I and II. We exclude patients with severe facial asymmetry or temporo-mandibular disorder. Written consents were obtained from the subjects, and the study was conducted according to the Declaration of Helsinki. Appropriate institutional review boards approved the study protocol (document ver. KHD IRB 1311-2).

**Table 1:** Comparison of tooth length changes between pre-treatment and post-treatment in each group

	T0	T1	T0-T1 differences	Range (95% confidence interval)		*P* value
	Mean ± SD	Mean ± SD	Mean ± SD	Min	Max	
Group A	26.07 ± 1.71	24.46 ± 1.52	1.61 ± 0.45	1.35	1.86	.000**
Group B	25.07 ± 2.26	24.11 ± 2.12	0.96 ± 0.66	0.58	1.35	.000**

** *P* <0.01

Group A (14 subjects): interdental space closure using traditional orthodontic treatment after maxillary first premolar extractions.

Group B (14 subjects): interdental space closure using anterior segmental osteotomy with maxillary first premolar extractions.

### Cone-beam computed tomographic and cephalometric analysis

**Fig. 1:**
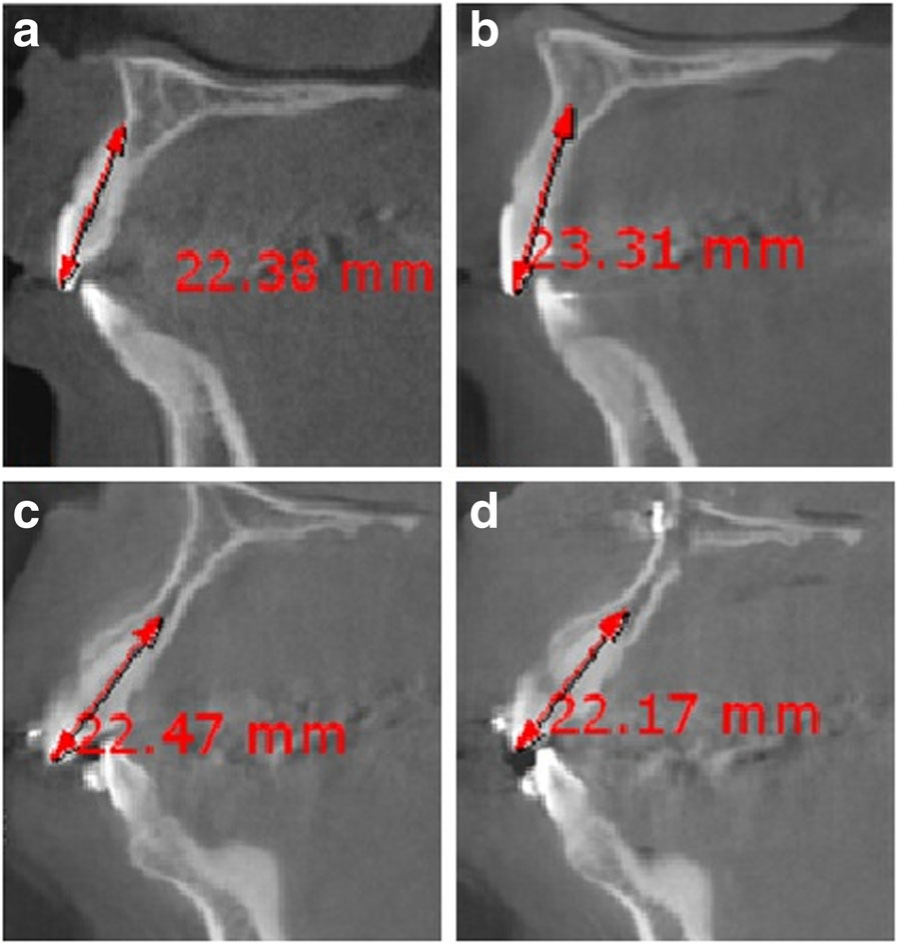
Measurement of tooth length on CBCT image of group A on T0 (**a**) and T1 (**b**) and group B on T0 (**c**) and T1 (**d**)

Cone-beam computed tomographic and cephalometric evaluations were retrospectively performed for the diagnosis of the treatment before treatment (T0) and at the end of active treatment (T1). The tooth lengths were assessed with cone-beam computed tomography (CBCT) in panoramic mode (Fig. 1.). An alphard-Vega 3030 Dental CT system (Asahi Roentgen Ind. Co., Ltd., Kyoto, Japan) was used in this study for CBCT scan. The subjects’ head was positioned and placed in a head holding device to ensure the FH plane was parallel to the floor. Scan conditions included a tube voltage of 80 kVp, tube current of 5 mA, and exposure time of 17 s. Lateral cephalography was performed for the assessment of anteroposterior position and incisal angle, with the orbital-auricular plane (F-H plane) parallel to the floor and the subject in the upright position at a 165-cm focal film distance and a 15-cm film distance from the sagittal plane using the CX 90SP (Asahi, Tokyo, Japan; < 70 kVp, 100 mA).

**Fig. 2:**
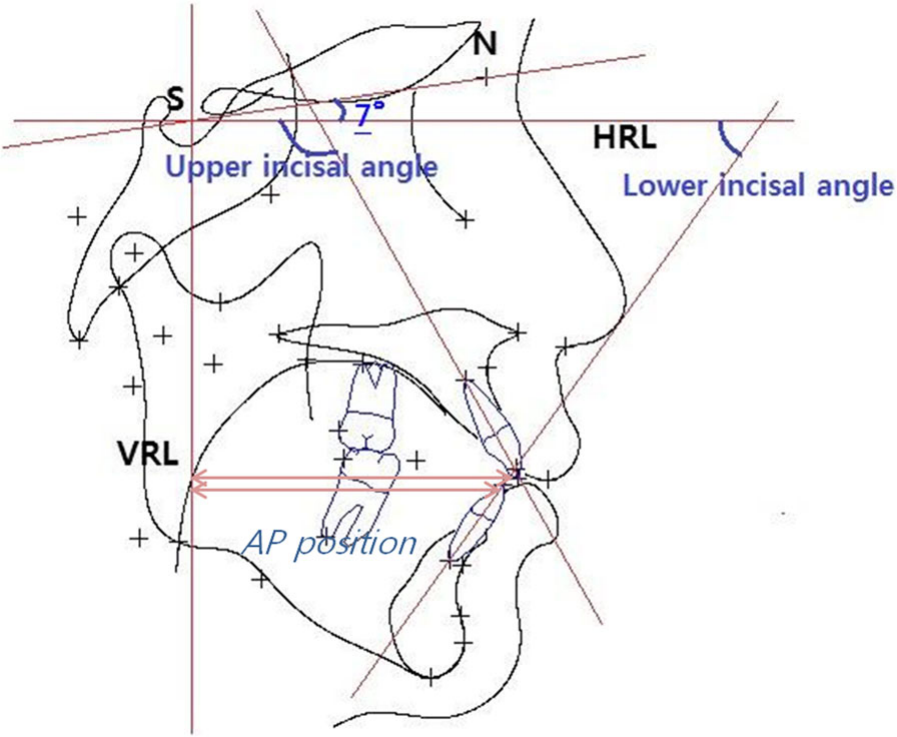
Landmarks: S: sella, N: nasion, U: upper incisor tip, L: lower incisor tip, VRL, HRL, incisor angle, AP position. Horizontal reference line (HRL): a line drawn 7° clockwise from the sella-nasion line with sella as the center. Vertical reference line (VRL): a line perpendicular to the HRL through the sella. Tooth length: distance from upper or lower incisor tip to apex. Incisal angle: an angle between U1 or L1 line (line through with tip and apex of central upper incisor) with HRL. AP position: distance from upper or lower incisor tip to VRL

Cephalometric landmarks and measurements are shown in Fig. 2 and corresponding measurements detailed in Table 2. Cephalometric analysis was traced by single examiner using V-ceph program Version 4.0 (CYBERMED Inc., Seoul, Korea).

**Table 2:** Comparison of the change between two groups

Mean (Δ = T0-T1)	Group A	Group B	*P* value
	Mean ± SD	Mean ± SD	
ΔRoot resorption (mm)	1.61 ± 0.45	0.93 ± 0.62	.006**
ΔRoot resorption (%)	6.13 ± 1.53	3.66 ± 2.39	.008**
ΔAngle (°)	11.93 ± 6.48	7.18 ± 5.33	.044*
ΔAP position (mm)	7.43 ± 1.93	7.57 ± 2.39	.863
ΔDuration (months)	28.20 ± 4.68	22.05 ± 5.99	.005**

*P < 0.05, **P < 0.01

### Statistical analysis

For statistical analysis of pre- and post-treatment changes, initially descriptive statistics such as mean and standard deviation were processed. The assumption of normality of the data was tested by Shapiro-Wilks tests. As the assumption of normality of the data has been rejected about the amount and ratio of root resorption, the Mann-Whitney *U* test was used for the above two items; the two sample *t* tests were used for the others. To test their significance for pre- and post-treatment changes, paired *t* tests were conducted. A significance level of 0.05 was predefined in all cases. To analyze the correlation between the variables within the groups, Pearson bivariate correlation analysis was conducted. A software package (SPSS 18.0, Chicago, IL, USA) was used for the statistical analysis.

## Results

### Comparison of changes between pre- and post-treatment in each group (Table 1)

In group A, the tooth length decreased from 26.07 ± 1.71 at T0 to 24.46 ± 1.52 at T1. In group B, the tooth length decreased from 25.07 ± 2.26 at T0 to 24.11 ± 2.12 at T1. There was a statistically significant difference between T1 and T0 within each group.

### Comparison of the change between two groups (Table 2)

Group B exhibits 0.93 mm of root resorption; this value is significantly lower than that of group A. The change of incisal angle (ΔAngle (°)) is significantly smaller in group B (7.18°) than in group A (11.93°). No significant changes were found in AP position between group A (7.43 mm) and group B (7.57 mm). The orthodontic treatment duration was significantly shorter for group B (22.05 months), compared to group A (28.2 months).

### Correlation between variables in each group (Table 3)

#### Group A

The change of incisal angle (ΔAngle (°)) was positively correlated with the change of AP position. Negative correlation was showed between the change of incisal angle (ΔAngle (°)) and the orthodontic treatment duration, and between change of AP position and the orthodontic treatment duration. No correlation was showed among other variables.

**Table 3:** The correlation between variables in each group

Significance/correlation coefficient	Δ Root resorption (mm)	Δ Root resorption (%)	Δ Angle (°)	Δ AP position (mm)	Δ Duration (months)
Group A (Pearson’s correlation)					
ΔRoot resorption (mm)	–	.000**	.918	.905	.630
	1	.980^b^	− .030	− .035	− .141
ΔRoot resorption (%)	.000**	–	.987	.899	.584
	.980^b^	1	− .005	− .038	− .160
ΔAngle (°)	.918	.987	–	.002**	.024*
	− .030	− .005	1	.756^b^	− .596^a^
ΔAP position (mm)	.905	.899	.002**	–	.016*
	− .035	− .038	.756^b^	1	− .627^b^
ΔDuration (months)	.630	.584	.024*	.016*	–
	− .141	− .160	− .596^a^	− .627^b^	1
Group B (Spearman’s correlation)					
ΔRoot resorption (mm)	–	.000**	.313	.498	.024*
	1	.963^b^	.280	.190	.577^a^
ΔRoot resorption (%)	.000	–	.248	.697	.063
	.963^b^	1	.318	.110	.491
ΔAngle (°)	.313	.248	–	.009**	.680
	.280	.318	1	.646^b^	.116
ΔAP position (mm)	.498	.697	.009**	–	.414
	.190	.110	.646^b^	1	.228
ΔDuration (months)	.024*	.063	.680	.414	–
	.577	.491	.116	.228	1

*P < 0.05, **P < 0.01

Mild correlation

^a^Moderate correlation

^b^High correlation

#### Group B

The amount of root resorption (mm) was positively correlated with the orthodontic treatment duration. The change of incisal angle (ΔAngle (°)) was positively correlated with the change of AP position. No correlation was showed among other variables.

## Discussion

Root resorption is prone to occur in patients with orthodontic treatment [13], but because of the presence of several complicated root resorption-related factors, it is difficult to provide accurate reasons [14]. Among these, known patient-related factors include genetic factors [15–18]; root resorption or traumatic anamnesis [17, 19]; anamnesis of root canal treatment [19, 20]; anatomical factors such as shape or length of the tooth root [16, 21–23], contiguity between the roots of teeth and the cortical bone [24, 25], and density of the alveolar bone [16, 24]; the degree of malocclusion [16, 17, 21, 26]; and patient age [21, 27] and gender [16, 19, 22]. In addition, orthodontic treatment-related factors include treatment duration [22–24], strength of the force applied [28], force application method (continuous force or intermittent force) [20, 25, 27], direction of tooth movement [28, 29], displacement of the tooth root [22, 26], and the type of orthodontic appliance [30]. Root resorption is affected by the complex functions of these various factors.

In a study on the degree of root resorption evaluated using periapical radiographs, over 1.4 mm of root resorption was reported in the maxillary anterior teeth [16], and Blake et al. reported 6 to 13% root resorption on study using periapical radiographs [31]. In addition, Brin et al. observed at least mild root resorption in approximately 80% maxillary incisors and moderate to severe root resorption in more than 12% [21]. Levander et al. and Taithongchai et al. reported that 1–5% teeth exhibited root resorption, with severe resorption defined as more than 4 mm or a third of the original tooth root [32, 33].

In the present study, group A underwent space closure by conventional orthodontic treatment only and showed 1.61 mm of root resorption, similar to the results of Sameshima et al. On the other hand, group B received ASO and exhibited 0.93 mm of root resorption; this value was significantly lower than the A group. These results show lesser root resorption with ASO than with conventional orthodontic treatment, which also supports the claim of many researchers who mentioned the benefits of ASO in terms of root resorption. As mentioned previously, ASO is a favorable treatment option when the possibility of root resorption occurrence is expected to be high due to risk factors such as anamnesis of trauma, anatomical limitations, and root contiguity with the cortical bone.

The treatment duration was also significantly shorter in group B (22.05 months) than in group A (28.2 months), suggesting that the treatment duration can be decreased when ASO is performed. Lee et al. and Kim et al. reported that ASO is a more attractive treatment option for adult patients because the treatment period is shorter and the improvement in facial form is immediate [12, 34].

Soft tissue improvements are also more superior with ASO and orthodontic treatment than with conventional orthodontic treatment only. Conventional orthodontic treatment only tends to result in retroclination of the anterior teeth, whereas ASO minimizes this and enables the bodily movement of teeth. In addition, the former increases tooth exposure by downward shifts of the maxillary anterior teeth, increasing gingival visibility and a gummy smile. In contrast, ASO resolves a gummy smile by facilitating vertical adjustments in tooth position [12].

However, ASO also has adverse effects such as necrosis of the anterior fragment, increase of the nasal base width, counter-clockwise rotation of the nasal tip, tooth root cutting, and spasticity of the canine, cleavage of the osteotomy segment, and discordance between the canine and the occlusal plane of the premolar tooth [35]. Several approaches have been attempted to overcome these limitations. Since the introduction of maxillary ASO by Cohn-stock in 1921, it has been performed using three methods in general, namely the Wassmund method, the Wunderer method, and the Cupar and Epker method [36]. The surgical method used in the present study was a modified Wassmund method (Lee’s method). After the placement of labial vertical incisions in the region of the premolars on both sides and a vertical incision in the median segment, tunneling was performed, followed by the osteotomy from both premolar regions up to the piriform aperture. Horizontal osteotomy was performed via a palatal approach through the region of the labial osteotomy. In particular, since it was difficult to access to the center of the labial segment via the conventional Wassmund method, we placed a labial vertical incision in the maxillary median segment to facilitate access, and blood supply to the labial and palatal mucosa was maintained. Through these methods, avascular necrosis of the segmental osteotomy region was prevented.

Recently, delicate traction, indentation, or extrusion of the anterior teeth by modifications of conventional orthodontic treatment methods using anchorage devices such as improved mini-screws or mini-plates have become possible. Therefore, the rate of selection of ASO as a treatment option has decreased among orthodontists. However, the limitations of conventional orthodontic treatment still exist, such as longer treatment duration, inability to correct severe facial deformities, possibility of excessive orthodontic force on the teeth, and possibility of anchorage loss due to anatomical limitations [37].

## Conclusions

In present study, the results show lesser root resorption and treatment duration with ASO than with conventional orthodontic treatment. Therefore, if the indications are accurately determined, ASO can be an effective treatment option when the possibility of root resorption occurrence is expected to be high because of risk factors such as anamnesis of trauma, anatomical limitations, and root contiguity with the cortical bone, especially in late adults.

## Abbreviations

AP: Anteroposterior
ASO: Anterior segmental osteotomy
CBCT: Cone-beam computed tomography

## References

[1] Lew KK, Loh FC, Yeo JF, Loh HS (1989). Profile changes following anterior subapical osteotomy in Chinese adults with bimaxillary protrusion. Int J Adult Orthodon Orthognath Surg..

[2] Baek SH, Kim BH (2005). Determinants of successful treatment of bimaxillary protrusion: orthodontic treatment versus anterior segmental osteotomy. J Craniofac Surg.

[3] Yokoo S, Komori T, Watatani S (2003). Indications and procedures for segmental dentoalveolar osteotomy: a review of 13 patients. Int J Adult Orthodon Orthognath Surg..

[4] Shawky MM, El-Ghareeb TI, Hameed Abu Hummos LA (2012). Evaluation of the three-dimensional soft tissue changes after anterior segmental maxillary osteotomy. Int J Oral Maxillofac Surg.

[5] Cohn-Stock G (1921). Die chirurgische immediatre-gulierung der Keifer, Speziell die chirurgische Behandlung der Prognathie. Vjschr Zahnheilk Berlin.

[6] Wassmund M (1935). Lehrbuch der praktischen chirurgie des Mundes under Keifer. Leipzig: Barth.

[7] Cupar I (1954). Surgical treatment of alterations in form and position of the maxilla. Osterr Z Stomatol.

[8] Schuchardt K (1955). Formen des offenen Bisses und ihre operative Behandlungsmoeglichkeiten. Fortschr Kieferorthop.

[9] Wunderer S (1963). Erfahrungen mit der Operativen Behandlung hochgradiger Prognathien. Dtsch Zahn-Mund-Kieferheilk.

[10] Bell WH, Condit CL (1970). Surgical-orthodontic correction of adult bimaxillary protrusion. J Oral Maxillofac Surg.

[11] Park JU, Hwang YS (2008). Evaluation of the soft and hard tissue changes after anterior segmental osteotomy on the maxilla and mandible. J Oral Maxillofac Surg.

[12] Lee JK, Chung KR, Baek SH (2007). Treatment outcomes of orthodontic treatment, corticotomy-assisted orthodontic treatment, and anterior segmental osteotomy for bimaxillary dentoalveolar protrusion. J Plast Reconstr Aesthet Surg.

[13] Killiany DM (1999). Root resorption caused by orthodontic treatment: an evidence-based review of literature. Semin Orthod.

[14] Weltman B, Vig KW, Fields HW, Shanker S, Kaizar EE (2010). Root resorption associated with orthodontic tooth movement: a systematic review. Am J Orthod Dentofac Orthop.

[15] Al-Qawasmi RA, Hartsfield JK, Everett ET (2003). Genetic predisposition to external apical root resorption. Am J Orthod Dentofac Orthop.

[16] Sameshima GT, Sinclair PM (2001). Predicting and preventing root resorption: part I. Diagnostic factors. Am J Orthod Dentofac Orthop.

[17] Hartsfield JK, Everett ET, Al-Qawasmi RA (2004). Genetic factors in external apical root resorption and orthodontic treatment. Crit Rev Oral Biol Med.

[18] Ngan DC, Kharbanda OP, Byloff FK, Darendeliler MA (2004). The genetic contribution to orthodontic root resorption: a retrospective twin study. Aust Orthod J.

[19] Drysdale C, Gibbs SL, Ford TR (1996). Orthodontic management of root-filled teeth. Br J Orthod.

[20] Brezniak N, Wasserstein A (2002). Orthodontically induced inflammatory root resorption. Part II: the clinical aspects. Angle Orthod.

[21] Brin I, Tulloch JF, Koroluk L, Philips C (2003). External apical root resorption in class II malocclusion: a retrospective review of 1- versus 2-phase treatment. Am J Orthod Dentofac Orthop.

[22] Fox N (2005). Longer orthodontic treatment may result in greater external apical root resorption. J Evid Based Dent Pract.

[23] Sameshima GT, Sinclair PM (2004). Characteristics of patients with severe root resorption. Orthod Craniofac Res..

[24] Otis LL, Hong JS, Tuncay OC (2004). Bone structure effect on root resorption. Orthod Craniofac Res.

[25] Horiuchi A, Hotokezaka H, Kobayashi K (1998). Correlation between cortical plate proximity and apical root resorption. Am J Orthod Dentofac Orthop.

[26] Segal GR, Schiffman PH, Tuncay OC (2004). Meta analysis of the treatment-related factors of external apical root resorption. Orthod Craniofac Res.

[27] Linge L, Linge BO (1991). Patient characteristics and treatment variables associated with apical root resorption during orthodontic treatment. Am J Orthod Dentofac Orthop.

[28] Barbagallo LJ, Jones AS, Petocz P, Darendeliler MA (2008). Physical properties of root cementum: Part 10. Comparison of the effects of invisible removable thermoplastic appliances with light and heavy orthodontic forces on premolar cementum. A microcomputed-tomography study. Am J Orthod Dentofac Orthop.

[29] Parker RJ, Harris EF (1998). Directions of orthodontic tooth movements associated with external apical root resorption of the maxillary central incisor. Am J Orthod Dentofac Orthop.

[30] Pandis N, Nasika M, Polychronopoulou A, Eliades T (2008). External apical root resorption in patients treated with conventional and self-ligating brackets. Am J Orthod Dentofac Orthop.

[31] Blake M, Woodside DG, Pharoah MJ (1995). A radiographic comparison of apical root resorption after orthodontic treatment with the edgewise and speed appliances. Am J Orthod Dentofac Orthop.

[32] Levander E, Malmgren O (1988). Evaluation of the risk of root resorption during orthodontic treatment: a study of upper incisors. Eur J Orthod.

[33] Taithongchai R, Sookkorn K, Killiany DM (1996). Facial and dentoalveolar structure and the prediction of apical root shortening. Am J Orthod Dentofac Orthop.

[34] Kim JR, Son WS, Lee SG (2002). A retrospective analysis of 20 surgically corrected bimaxillary protrusion patients. Int J Adult Orthodon Orthognath Surg..

[35] Lew KK (1991). Orthodontic considerations in the treatment of bimaxillary protrusion with anterior subapical osteotomy. Int J Adult Orthodon Orthognath Surg.

[36] Epker BN (1977). A modified anterior maxillary ostectomy. J Maxillofac Surg.

[37] Yamaguchi M, Inami T, Ito K, Kasai K, Tanimoto Y (2012). Mini-implants in the anchorage armamentarium: new paradigms in the orthodontics. Int J Biomater.

